# Update on the Genetic Polymorphisms of Drug-Metabolizing Enzymes in Antiepileptic Drug Therapy

**DOI:** 10.3390/ph3082709

**Published:** 2010-08-20

**Authors:** Junji Saruwatari, Takateru Ishitsu, Kazuko Nakagawa

**Affiliations:** 1 Division of Pharmacology and Therapeutics, Graduate School of Pharmaceutical Sciences, Kumamoto University, Oe-honmachi 5-1, Kumamoto 862-0973, Japan; E-Mail: junsaru@gpo.kumamoto-u.ac.jp (J.S.); 2 Kumamoto Saishunso National Hospital, Kumamoto, Suya 2659, Koshi, Japan; E-Mail: ishitsu@bf7.so-net.ne.jp (T.I.); 3 Center for Clinical Pharmaceutical Sciences, Kumamoto University, Oe-honmachi 5-1, Kumamoto 862-0973, Japan

**Keywords:** pharmacogenetics, genetic polymorphisms, antiepileptic drugs, drug-metabolizing enzyme, population pharmacokinetics

## Abstract

Genetic polymorphisms in the genes that encode drug-metabolizing enzymes are implicated in the inter-individual variability in the pharmacokinetics and pharmaco-dynamics of antiepileptic drugs (AEDs). However, the clinical impact of these polymorphisms on AED therapy still remains controversial. The defective alleles of *cytochrome P450 (CYP) 2C9* and/or *CYP2C19* could affect not only the pharmacokinetics, but also the pharmacodynamics of phenytoin therapy. *CYP2C19* deficient genotypes were associated with the higher serum concentration of an active metabolite of clobazam, *N*-desmethylclobazam, and with the higher clinical efficacy of clobazam therapy than the other *CYP2C19* genotypes. The defective alleles of *CYP2C9* and/or *CYP2C19* were also found to have clinically significant effects on the inter-individual variabilities in the population pharmacokinetics of phenobarbital, valproic acid and zonisamide. *EPHX1* polymorphisms may be associated with the pharmacokinetics of carbamazepine and the risk of phenytoin-induced congenital malformations. Similarly, the *UDP-glucuronosyltransferase 2B7* genotype may affect the pharmacokinetics of lamotrigine. *Gluthatione S-transferase* null genotypes are implicated in an increased risk of hepatotoxicity caused by carbamazepine and valproic acid. This article summarizes the state of research on the effects of mutations of drug-metabolizing enzymes on the pharmacokinetics and pharmacodynamics of AED therapies. Future directions for the dose-adjustment of AED are discussed.

## 1. Introduction

Epilepsy is a disorder of the brain characterized by an enduring predisposition to generate epileptic seizures, and epileptogenesis is the development of a neuronal network in which spontaneous seizures occur [1,2,3]. Epilepsy is a common chronic neurological disease, of which the worldwide prevalence is estimated to be 0.6–1.0% [1,2,3].

The treatment outcome for epilepsy with antiepileptic drugs (AEDs) may vary, even between patients with seemingly the same epilepsy syndrome [1,4]. It is estimated that 20–25% of patients with epilepsy fail to achieve good control with an AED [1,4,5]. Although the incidence of adverse effects differs among AEDs, it is currently not possible to predict the efficacy and adverse effects in an individual patient [6,7,8,9]. Some of the observed variability in individual responses to AEDs depends on well-known factors that are easily assessable, such as age, sex, weight, liver and renal function, co-medication, heterogeneity in the disease, nutritional state, and/or smoking status [10,11]. However, further differences in the response may, at least in part, result from the genetic variation in the patients [5,6,7,8,9,12].

Pharmacogenetics concerns the link between the genetic constitution of an individual and his/her reaction to drugs [10,13,14,15,16]. Although this discipline promises the possibility of one day choosing the right drug for any individual, *i.e.*, effective treatment without major adverse effects at an effective dose, only a few of isolated examples has been available in clinical practice [10,15,16]. Among the AED therapies, the individual patient genotype influences almost all stages of pharmacokinetics and pharmacodynamics [6,7,9,17,18,19,20].

The topic of pharmacogenetics in epilepsy has previously been covered in several excellent review articles [5,8,21,22,23]. In a review published in 2009, the impact of genetic variations in drug-metabolizing enzymes was concluded to have a limited clinical impact on AED therapy [7]. The aim of the present article was to provide a more recent update on the clinical impact of genetic polymorphisms of drug-metabolizing enzymes on the pharmacokinetics and pharmacodynamics of AEDs. Future directions for the AED dose-adjustment according to both genetic and non-genetic factors affecting the pharmacokinetics of AEDs are also discussed.

## 2. Search Strategy and Selection Criteria

We identified articles through a system search of the Ovid MEDLINE database and through reference lists of selected articles up to April 30, 2010. The following terms were used for the MEDLINE search: (*epilepsy* [MeSH terms] OR *anticonvulsants* [MeSH terms] OR *antiepileptic drug* [All Fields] OR *carbamazepine* [All Fields] OR *clobazam* [All Fields] OR *clonazepam* [All Fields] OR *ethosuximide* [All Fields] OR *felbamate* [All Fields] OR *gabapentin* [All Fields] OR *lamotrigine* [All Fields] OR *levetiracetam* [All Fields] OR *oxcarbazepine* [All Fields] OR *phenobarbital* [All Fields] OR *phenytoin* [All Fields] OR *primidone* [All Fields] OR *topiramate* [All Fields] OR *valproic acid* [All Fields] OR *valproate* [All Fields] OR *zonisamide* [All Fields]) AND (*pharmacokinetics* [MeSH terms] OR *drug-metabolizing enzyme* [All Fields] OR *cytochrome P450* [All Fields] OR *epoxide hydrolase* [All Fields] OR *glucuronosyltransferase* [All Fields] OR *glutathione transferase* [All Fields]) AND (*pharmacogenetics* [All Fields] OR *pharmacogenomics* [All Fields] OR *genetic* [All Fields] OR *polymorphism* [All Fields] *genotype* [All Fields] OR *allele* [All Fields] OR *mutation* [All Fields]). Articles published between 1950 and 2010 were included. No language or ethnic restriction was applied for the search and study inclusion. We largely selected publications appeared in the past five years, but did not exclude commonly referenced and highly regarded older publications. Review articles are cited to provide readers with more details and references than are provided in this article.

## 3. Pharmacokinetics of AEDs

Older AEDs such as phenytoin, carbamazepine, valproic acid (sodium valproate) and phenobarbital are characterized by a narrow therapeutic range and a pronounced inter-individual variability in their pharmacokinetics [11,22,24]. It is often claimed that the new generation of AEDs such as gabapentin and levetiracetam have a major advantage over the older AEDs in that they have more predictable pharmacokinetics [11,25]. However, for some of newer AEDs such as clobazam, lamotrigine, topiramate and zonisamide, pharmacokinetic variability is just as prevalent as for many of the older AEDs [22,25,26]. Individualization of dosing has therefore been a key concept in the effort to optimize drug treatment of epilepsy, and measuring drug concentrations in serum or plasma via therapeutic drug monitoring has become a major focus of clinical research and practice [11,25].

Table 1 summarizes the pharmacokinetic characteristics of the various AEDs. Elimination of drugs, including AEDs, is accomplished by the hepatic (metabolism) and/or renal (excretion) route (Table 1) [7,9,18,19]. Since the metabolism of AEDs represents the prominent pathway of elimination (Table 1), both in qualitative and quantitative terms, the inter-individual capacity of hepatic metabolism of AEDs is the primary cause of the variability in the pharmacokinetics, and even in the pharmacodynamics (*i.e.*, efficacy, tolerability and safety) of the AEDs [18,19].

## 4. Genetic Polymorphisms of Drug-Metabolizing Enzymes

### 4.1. Cytochrome P450 (CYP) superfamily

The CYP enzymes are encoded by 57 human genes whose products are involved in oxidative drug metabolism, as well as the synthesis of cholesterol, steroids, prostacyclins and thromboxanes. In the last few years, CYP enzymes have attracted increasing attention, and it has been demonstrated that many CYPs have genetic polymorphisms [27,28,29]. Most AEDs, except for gabapentin, lamotrigine and levetiracetam, are metabolized at least partially by CYP enzymes (Table 1) [18]. Therefore, genetic polymorphisms of *CYP* enzymes are implicated in the inter-individual variability in the pharmacokinetics of AEDs [7,12,17,18,19]. 

**Table 1:** Pharmacokinetic characteristics of antiepileptic drugs.

AED	Oral bioavailability	t_1/2_ (hour)	Clearance	Active metabolites	Enzymes involved in the metabolism of the compound
Carbamazepine	75*–*85%	12*–*24	>95% Hepatic	Carbamazepine-10,11-epoxide	CYP3A4/5, CYP2C8, mEH, UGTs
Clobazam	87%	22*–*30	>95% Hepatic	*N*-desmethylclobazam	CYP3A4 and CYP2C19
Clonazepam	>80%	19*–*60	>95% Hepatic	—	CYP3A4
Ethosuximide	<100%	36*–*60	65% Hepatic	—	CYP3A4
			35% Renal		
Felbamate	90%	14*–*23	50% Hepatic	—	CYP2C19?, UGTs
			50% Renal		
Gabapentin	45*–*70%	5*–*7	100% Renal	—	None
Lamotrigine	<100%	24*–*36	90% Hepatic	—	UGTs
			10% Renal		
Levetiracetam	<100%	6*–*8	66% Renal	—	Nonhepatic hydrolysis (in blood)
			34% Hepatic		
Oxcarbazepine	> 95%	1*–*2	45% Hepatic	MHD	UGTs
			65% Renal		
Phenobarbital	80*–*100%	72*–*96	75% Hepatic	—	CYP2C19, CYP2C9
			25% Renal		
Phenytoin	95%	20*–*50	>90% Hepatic	—	CYP2C9, CYP2C19
Primidone	<100%	10*–*20	50% Hepatic	Phenobarbital	CYP2C9 (for phenobarbital)
			50% Renal	Phenylethylmalonamide	
Topiramate	80%	20*–*30	30*–*50% Hepatic	—	CYP3A4, UGTs
			50*–*70% Renal		
Valproic acid	<100%	8*–*16	>95% Hepatic	—	UGTs, CYP2C9, CYP2C19
Zonisamide	<100%	50*–*70	>90% Hepatic	—	CYP3A4, CYP2C19, UGTs

This table is prepared based on the previous review articles [7,22,25,26] with modifications. AED, antiepileptic drug; t_1/2_, elimination half-life; CYP, cytochrome P450; mEH, microsomal epoxide hydrolase; UGTs, UDP-glucuronosyltransferases; MHD, monohydroxylated active metabolite of oxycarbazepin.

CYP2C9 and CYP2C19 have well-characterized functional variants. The frequencies of defective *CYP2C9* alleles (CYP2C9*2 and CYP2C9*3) are higher in Caucasians (18.9%) than in Asians (2.5–3.5%) [30,31]. Conversely, the frequencies of defective *CYP2C19* alleles (CYP2C19*2 and CYP2C19*3) are higher in Asians (33–43.5%) than Caucasians (13.6%) [30,31,32]. Population studies have shown that individuals can be classified into three subgroups: homozygous extensive metabolizers (homo EMs), heterozygous EMs (hetero EMs) and poor metabolizers (PMs) according to the number (*i.e.*, 0, 1 and 2, respectively) of the defective alleles(s) of each *CYP2C* gene [12,30,31,32]. Individuals with defective alleles of the *CYP2C9* or *CYP2C19* genes have been shown to have a reduced metabolism of some AEDs compared with those with wild-type (normal) alleles [7,17,18,19,33,34,35,36,37,38,39]. 

In addition to CYP2C9/2C19, CYP3A5 is polymorphically expressed in the liver, small intestine and kidney, and represents 5 to 85% of the total hepatic and intestinal CYP3A4/5 content [40,41,42]. The most common *CYP3A5* polymorphism is CYP3A5*3, which has a frequency of 65*–*85% in Asians, 84–95% in Caucasians and 27*–*55% in African-Americans [30,31,40,41,42]. This allele contains a splice variant, which encodes a truncated nonfunctional protein [40,41,42]. Regardless of race, *in vitro* studies using human liver and intestine preparation have demonstrated that homozygosity for the CYP3A5*3 allele leads to decreased expression of *CYP3A5* mRNA and protein in comparison with the heterozygous or homozygous wild-type (CYP3A5*1) allele [40,41,42]. However, the clinical importance of CYP3A5*3 polymorphisms in pharmacokinetics of AEDs is not yet clear [38,43,44].

### 4.2. Microsomal epoxide hydrolase

Oxidation by one or more of the phase I oxidative enzymes such as the CYP superfamily often results in the formation of reactive xenobiotic epoxide [45]. The microsomal epoxide hydrolase (mEH) encoded by *EPHX1* is a biotransformation enzyme that metabolizes numerous reactive epoxide intermediates to more water-soluble trans-dihydrodiol derivatives [45]. Human mEH expression has been demonstrated in all of the tissues examined, with highest levels in the liver, kidney and testes [45,46,47] and 10- to 100-fold lower levels in the lungs and lymphocytes [48].

Marked inter-individual variations in mEH activity have been reported [49]. These variations may be due to genetic polymorphisms in the coding regions as well as in the promoter region of the *EPHX1* [45,50,51,52,53]. The two nonsynonymous polymorphisms, Try113His and His139Arg, are common in Caucasians as well as Asians. The allele frequency of Try113His (337T>G) is reported to be 22–31% in Caucasians and approximately 45% in Asians, and that of His139Arg (416A>G) is 20–22% in Caucasians and approximately 14% in Asians [54,55,56]. Early *in vitro* expression studies demonstrated that the Try113His variant confers a nearly 40% decrease in hydrolase activity, whereas the His139Arg variant confers an increase in activity of at least 25% [49]. These variant alleles have also been reported to affect enzyme stability [57]. These polymorphisms in *EPHX1* could therefore affect the pharmacokinetics of AEDs and the fetal exposure to the reactive oxide intermediates in pregnant women with these polymorphisms [45,58,59,60,61].

### 4.3. Uridine diphospho-glucuronosyltransferase (UGT)

UGT enzymes are key metabolic proteins that prevent the accumulation of potentially toxic lipophilic compounds and initiate their elimination through more the hydrophilic biliary and renal systems [62]. This is accomplished by the addition of a hydrophilic sugar moiety (glucuronide) from uridine diphosphate (UDP) glucuronic acid by UGTs [62]. The UGT enzymes have been classified into two major families in humans, namely UGT1A and UGT2 (subdivided into UGT2A and UGT2B) [62]. The influence of inheritable polymorphisms on human UGT-encoding genes has been extensively documented, and was shown to be responsible for a fraction of the observed phenotypic variability in metabolism and excretion [63]. 

Pathways involving members of the *UGT1* superfamily act on approximately 35% of all drugs metabolized by phase II drug metabolizing enzymes [64], including several AEDs such as carbamazepine, valproic acid, lamotrigine, oxcarbazepine, topiramate and zonisamide [22,25]. One of the common genetic polymorphisms in *UGT1A* genes is a TA insertion in the *UGT1A1* TATA-box, 41 nucleotides upstream of the translation start site, leading to the variant (TA)_7_ allele (UGT1A1*28) instead of the (TA)_6_ reference allele (UGT1A1*1). This *UGT1A1* promoter region dinucleotide repeat polymorphism results in reduced expression levels by altering transcription initiation, and also results in an approximately 70% reduction in glucuronidation of bilirubin and other UGT1A1 substrates [65,66]. A number of studies have shown that UGT1A1*28 is relatively frequent in many populations, with an allele frequency 32–39% in Caucasians, 40*–*43% in Africans, and 16*–*18% in Asians [65,66].

UGT2B7 also contributes to the glucuronidation of AEDs such as valproic acid, carbamazepine and lamotrigine [67,68,69]. Several polymorphisms have been identified within the *UGT2B7* gene. The A to T transversion at nucleotide 802 leads to a change in amino acid sequence, His268Try. This allele is designated as UGT2B7*2, and the frequency of the UGT2B7*2 allele is 48.9 and 26.8% in Caucasians and Asians, respectively [70]. Variants have also been identified within the *UGT2B7* promoter that exhibit some ethnic/race-dependent haplotype structures and seem to depend more on linkage to structural variants [53]. A haplotype defined by six promoter variants, involving -901G>A and -161T>C, was observed at a frequency between 44 and 55% in Caucasians and about 25% in Asians [71,72,73]. Functional analysis of this haplotype *in vitro* revealed an approximate 2-fold increase in activity in both HepG2 hepatoma cells and CaCo-2 colon cells [71].

These genetic polymorphisms may affect the pharmacokinetics of AEDs, thereby altering their efficacy and adverse event profiles [23]. However, in most instances, the functional significance of these polymorphisms is unclear for a number of reasons; isozyme substrate specificity remains poorly defined, isoforms may exhibit overlapping substrate specificity, and the domains of UGT proteins responsible for substrate binding have not been identified [62,74].

### 4.4. Glutathione S-transferase (GST)

GST catalyzes the conjugation of glutathione (GSH) with electrophiles, generally resulting in their detoxification and facilitated elimination [75]. The ability to conjugate electrophiles makes these enzymes critical in the detoxification of a wide range of epoxides and certain other agents, including therapeutic drugs, pesticides and dietary components [76,77]. Since some AEDs are biotransformed to reactive oxides during their metabolism, GST is one of the key enzymes in the detoxification of these metabolites [78]. 

Polymorphisms that exert substantial effects on the GST function have been noted in human populations for several isozymes [78]. Monte Carlo simulations indicated large inter-individual variability for GSTM1 and T1 due to the presence of the null (zero activity) genotype, which is common in all populations studied [78]. The *GSTM1* and *GSTT1* genotypes have also been studied across wide variety of populations [78]. The *GSTM1* null genotype is common, with frequencies of 40–60% in Caucasians and Asians, and 20–25% in African Americans [78]. The *GSTT1* null genotype is less frequent than the *GSTM1* null genotype in Caucasians (18%) but has a similar frequency to the *GSTM1* null genotype in Asian groups (generally ranging from 40*–*60%) and in African Americans (22%) [78]. Several studies also reported the double null genotype (*GSTM1* null and *GSTT1* null) with frequencies close to what is expected by assuming independent inheritance and thus simply multiplying the two null frequencies together. The double null frequency was reported to occur at frequency of 9.6% in Caucasians, 6% in African Americans, 20–33% in Asians [78].

## 5. Effect of Genetic Polymorphisms of Drug-Metabolizing Enzymes on AED Therapy

### 5.1. Carbamazepine

Carbamazepine is extensively metabolized in the liver, with less than 5% of an oral dose excreted unchanged in urine [79]. Carbamazepine is predominantly metabolized by CYP3A4, and partially by CYP2C8, to carbamazepine-10,11-epoxide. Carbamazepine-10,11-epoxide is subsequently transformed by mEH to the inactive carbamazepine-10,11-diol, which is finally excreted into the urine in free and conjugated forms [80]. Carbamazepine-10,11-epoxide and carbamazepine-10,11-trans-diol are the primary (≤60%) metabolites in urine [81,82]. Carbamazepine has a narrow therapeutic range between serum concentrations of 3–12 μg/mL [83].

#### 5.1.1. Pharmacokinetics

In a recent study of 35 Korean patients treated with carbamazepine monotherapy, patients with the CYP3A5*3/*3 genotype showed 31% higher serum levels of carbamazepine compared to those with CYP3A5*1/*1 or *1/*3 genotypes [44]. In addition, the oral clearance of carbamazepine in patients with the CYP3A5*3/*3 genotype was 29% lower than that of patients with the CYP3A5*1/*1 or CYP3A5*1/*3 genotype [44]. The influence of the *CYP3A5* genotype on the population pharmacokinetics of carbamazepine were also assessed in 144 Japanese patients treated with carbamazepine polytherapy in combination with enzyme-inducing AEDs, phenytoin and phenobarbital, as well as with carbamazepine monotherapy [12,43]. In this study, the population clearance estimates for carbamazepine indicated that there was an 8% increase in the serum concentration in patients with CYP3A5*3/*3 compared to those with CYP3A5*1/*1 or *1/*3, suggesting that the CYP3A5*3 genotypes may not be as important in the pharmacokinetics of carbamazepine in Japanese patients with epilepsy. However, further studies are necessary to elucidate underlying mechanism of these findings in larger and different ethnic populations.

Several *EPHX1* polymorphisms are associated with altered carbamazepine-10,11-epoxide metabolism [54]. The plasma carbamazepine-10,11-trans-diol to carbamazepine-10,11-epoxide ratios increased significantly, depending on the number of haplotype *2 bearing Try113His, whereas the ratios decreased in patients with His139Arg [54]. In a recent study of 70 patients with epilepsy in Scotland, a multivariate model incorporating patient age and *EPHX1* Try113His and His139Arg polymorphisms, revealed a significant association between the genotypes and the maintenance dose of carbamazepine [84].

#### 5.1.2. Pharmacodynamics

Cutaneous adverse drug reactions (cADR), ranging from a mild maculopapular exanthema to life-threatening severe cutaneous reactions such as Stevens-Johnson syndrome, are among the most common adverse effects of AEDs [8]. The FDA issued an alert and updated product labeling recommending genetic testing for *HLA-B* polymorphisms to predict carbamazepine-induced serious skin reactions in individuals of Asian descent [85]. In a search for genetically-determined abnormalities in carbamazepine metabolism in 91 Taiwanese patients, 278 single nucleotide polymorphisms, including *CYP3A4*, *CYP1A2* and *EPHX1*, and the major histocompatibility complex region, tumor necrosis factor-α and heat shock protein, were screened [86]. Although an association between HLA-B*1502 and cADR was found, no polymorphisms involved in the metabolism of carbamazepine were identified as being pathogenic in these patients [86]. 

AEDs-related hepatotoxicity can be caused by immune-mediated mechanisms or direct cytotoxic damage [9,87]. A retrospective study in Japanese patients with epilepsy implicated the *GSTM1* null genotype as a risk factor for carbamazepine-induced mild hepatotoxicity, whereas the *EPHX1* polymorphisms did not lead to any elevation of transaminases in 192 Japanese patients treated with carbamazepine [88].

### 5.2. Clobazam

More than 70% of the administered dose of clobazam is demethylated to yield *N*-desmethylclobazam, a pharmacologically active metabolite that reaches higher plasma concentrations than clobazam and may substantially contribute to the efficacy and toxicity of long-term clobazam therapy [89,90]. This demethylation is facilitated by CYP3A4, CYP2C19 and CYP2B6, and the subsequent inactivation of *N*-desmethylclobazam to 4’-hydroxynorclobazam is catalyzed primarily by CYP2C19 [91]. There is little information about the target serum concentration of clobazam and *N*-desmethylclobazam.

#### 5.2.1. Pharmacokinetics

A recent study of 110 Japanese patients with epilepsy demonstrated that the mean serum concentration of *N*-desmethylclobazam was nine times higher in *CYP2C19* PMs than in *CYP2C19* homo EMs, and the degree of elevation in the serum *N*-desmethylclobazam/clobazam concentration ratio was dependent on the number of defective alleles of *CYP2C19*. This indicates that the *CYP2C19* genotypes are associated with the serum concentration of the active metabolite of clobazam, *N*-desmethylclobazam [35].

#### 5.2.2. Pharmacodynamics

In this same study [35], the responder rate was significantly greater in *CYP2C19* PMs and *CYP2C19* hetero EMs than in *CYP2C19* homo EMs, with a gene-dose effect (65.2, 47.6 and 33.3%, respectively), and the adjusted odds ratio (95% confidence interval) of *CYP2C19* PMs *versus* *CYP2C19* homo EMs was 9.88 (2.47*–*39.56). The incidence adverse reactions, including drowsiness and dizziness, tended to be higher in *CYP2C19* PMs (64.0%) than in the *CYP2C19* hetero EMs or *CYP2C19* homo EMs (43.2 and 39.0%, respectively, *P* = 0.07) [35], and the incidence in the homo EMs was similar to those in Canadian or European studies [92,93,94]. The frequency of *CYP2C19* PMs varies across races, for example, 13–23% of Asians and 1–8% of Caucasians are estimated to be PMs [30,31]. Therefore, a variety of *CYP2C19* polymorphisms may affect the incidence of the adverse effects of clobazam. On the other hand, the *CYP2C19* genotypes were not associated with the tolerance. These results suggest that *CYP2C19* polymorphisms are associated with the clinical efficacy of clobazam therapy, but that they are not associated with tolerance to the drug [35].

### 5.3. Lamotrigine

Lamotrigine undergoes glucuronidation on the 2-nitrogen atom of the triazine ring, leading to an inactive quaternary ammonium-linked *N*-2-glucuronide [95]. Lamotrigine is mainly metabolized by UGT1A4 [95], but other UGTs such as UGT1A1 and UGT2B7 contribute to lamotrigine glucuronidation [69,96]. Patients treated with therapeutic doses have serum lamotrigine concentrations in the order of 2.5–15 μg/mL [25].

#### 5.3.1. Pharmacokinetics

A recent study of Spanish patients with epilepsy revealed that a significant association was found between the lamotrigine concentration and the dose (C/D) ratio and *UGT2B7* -161C >T polymorphisms, and co-medication with agents such as valproic acid and enzyme-inducing AEDs was responsible for most of the inter-individual variability in the lamotrigine C/D ratio (70%), followed by patient age (24%) and the *UGT2B7* -161C>T polymorphism (12%) [97].

#### 5.3.2. Pharmacodynamics

To the best of our knowledge, no studies have been undertaken to examine whether genetic polymorphisms in *UGT* genes affect the efficacy or safety of lamotrigine therapy.

### 5.4. Phenytoin

Phenytoin exhibits a non-linear relationship between doses and serum concentrations, and the therapeutic window is narrow, with a range usually between 5 and 25 μg/mL [83]. Seventy to 90% of the oral administrated dose of phenytoin is oxidized, mainly by CYP2C9, and to a minor extent by CYP2C19, to yield *S*-5-(4*p*-hydroxyphenyl)-5-phenylhydantoin (*p*-HPPH) in humans [98,99]. The relative contribution of CYP2C19 to phenytoin metabolism increases as phenytoin concentrations increase, leading to saturation of CYP2C9 [99].

#### 5.4.1. Pharmacokinetics

The CYP2C9*2, CYP2C9*3 and CYP2C19*2 alleles have all been shown to affect the phenytoin plasma concentration and toxicity [33,34,39,100,101,102,103]. Several studies of phenytoin have shown that genetic polymorphisms of *CYP2C9* and *CYP2C19* correlate with the dose needed by patients to control seizures [33,34,39,100,101]. In a retrospective analysis of 269 patients with epilepsy from the UK, the maximal dose of phenytoin was stratified according to the *CYP2C9* genotypes of patients [39]. Carriers of one or two defective *CYP2C9* alleles (*i.e.*, *CYP2C9* hetero EMs or PMs) apparently needed a 13% and 30% lower dose, respectively [39]. In another analysis of 169 patients with epilepsy from Taiwan, a clinically relevant decrease in the maximal rate of metabolism (V_max_) and intrinsic clearance was observed when the patients were *CYP2C9* hetero EMs in combination with *CYP2C19* hetero EMs or PMs [34]. Based on the calculated pharmacokinetic parameters, the authors recommended reducing the normal dosage of phenytoin (5*–*7 mg/kg/day) to 2*–*4 mg/kg/day for carriers of these genotypes [34].

Another recent study found that CYP2C19*2 was in complete linkage disequilibrium with CYP2C9*1B, containing two genetic variants in the extended promoter of *CYP2C9*; the -3089G>A and -2663delTG polymorphisms in Caucasians [104]. Among patients who did not carry the CYP2C9*2 or *3 alleles, the CYP2C9*1B haplotype was significantly associated with a reduced phenytoin maintenance dose, consistent with reduced phenytoin induction of CYP2C9 metabolism in CYP2C9*1B carriers [104]. A multivariate model that includes all *CYP2C9* genotype information in addition to gender, body weight, and genomic ancestry showed that nearly half of the variation in phenytoin maintenance dose can be explained by these predictors, with up to 10% of the variation in dose attributable to the CYP2C9*1B haplotype alone [104]. 

#### 5.4.2. Pharmacodynamics

Several case studies reported that phenytoin-induced central nervous system toxicities were observed when the patients were carriers of defective *CYP2C9* and/or *CYP2C19* allele(s) [12,105,106,107,108]. Concerning the development of cADRs or gingival hyperplasia during phenytoin therapy, it is not yet clear whether there is an association with *CYP2C9*/*2C19* polymorphisms [109,110,111]. A previous review article suggested that individual pharmacogenetic differences in *CYP2C9* and *EPHX1* may increase susceptibility to birth defects after fetal exposure to phenytoin [61]. Recently, the risk of phenytoin-induced craniofacial abnormalities in children was assessed in relation to known maternal functional polymorphisms of *CYP2C9* and *EPHX1* in the Collaborative Perinatal Project database [58]. The risk in the child was lower for the maternal *EPHX1* 113Try/139His haplotype than in other *EPHX1* haplotypes (odds ratio = 0.29), whereas the *CYP2C9* genotype was not related to any of the fetal endpoints assessed [58].

### 5.5. Phenobarbital

Phenobarbital is eliminated by a combination of renal excretion of the unchanged drug (25%), *N*-glucoside formation (25%) and CYP2C9- and/or CYP2C19-dependent oxidation (≤25%) [112,113]. However, independent data showing a substantial contribution of these CYPs on phenobarbital metabolism are currently missing. In patients treated with therapeutic doses, serum phenobarbital concentrations in the order of 10*–*40 μg/mL have been reported [83].

#### 5.5.1. Pharmacokinetics

The clearance of phenobarbital was shown to be 18.8% lower in *CYP2C19* PMs, compared to other *CYP2C19* genotypes, in 74 Japanese patients with epilepsy [114], in which carriers of the defective *CYP2C9* allele(s) were excluded. The influences of the *CYP2C9* and *CYP2C19* genotypes on the population clearance estimates for phenobarbital were also evaluated in 79 Japanese patients with epilepsy [12,36]. The phenobarbital clearance in patients with the *CYP2C9* hetero EMs was 48% lower than in those with the homo EMs (*P* <0.001), whereas no effect of the *CYP2C19* genotype on the phenobarbital clearance was observed [12,36].

#### 5.5.2. Pharmacodynamics

Individuals with reduced CYP2C9 activity, carriers of one or two defective *CYP2C9* allele(s), are more prevalent in Caucasians (35%) than in African-Americans (13%) and Asians (3.5%) [30,31]. The incidences of phenobarbital-related adverse reactions were reported to be higher in Caucasians (22*–*60%) than in African-Americans (3.2%) and Asians (1%) [115,116,117,118]. The lower frequency of the individuals with reduced CYP2C9 activity among Asians and African-Americans might be a possible explanation for the higher tolerability of phenobarbital in these races compared to Caucasians [12,36].

### 5.6. Valproic acid

Valproic acid shows high inter-individual variability in pharmacokinetics and pharmacodynamics and has a narrow therapeutic range; therefore, its plasma level needs to be carefully monitored during the course of therapy (50*–*100 μg/mL) [119]. The major metabolic pathways of valproic acid comprise glucuronidation, β-oxidation and ω-oxidation. 

#### 5.6.1. Pharmacokinetics

A recent population pharmacokinetic study in 287 Chinese patients with epilepsy showed that *CYP2C9* in combination with *CYP2C19* genotypes significantly influenced the pharmacokinetic variability of valproic acid, as quantified by population pharmacokinetic analysis [120]. UGT2B7 also contributes to the glucuronidation of valproic acid [68,121]. A notable but non-significant tendency toward an increase in the area under the curve of serum concentrations of valproic acid was found as the number of UGT2B7*2 alleles increased [67].

#### 5.6.2. Pharmacodynamics

Valproic acid has a direct toxic action, and susceptibility to valproic acid-induced hepatotoxicity may be enhanced by alterations in the metabolism of valproic acid [9]. It has been known for a long time that, in rare cases, valproic acid can cause liver damage, which is partially due to the CYP-catalyzed formation of the hepatotoxic metabolite, 4-ene-valproic acid [122]. From *in vitro* studies, it was concluded that CYP2C9 mediates the metabolic conversion of valproic acid to form 4-ene-valproic acid [123]. However, it remains unresolved as to whether *CYP2C9* polymorphisms could be responsible for the hepatotoxic potential to valproic acid. As valproic acid can cause oxidative stress in rats, which precedes the onset of hepatotoxicity, a CYP-independent mechanism might also be in effect [124]. An association between the common polymorphisms in the *GSTM1* and *GSTT1* null genes and an increase of γ-glutamyltransferase, which was proposed to be a surrogate marker for mild hepatotoxicity in the study, was reported in 149 valproic acid-treated Japanese patients with epilepsy [125]. 

### 5.7. Zonisamide

Zonisamide is eliminated via renal excretion of the 2-sulfamoylacetyl-phenol-glucuronide (SMAP) (50%), of the unchanged form (35%) and of *N*-acetyl zonisamide (15%) [126,127]. The formation of SMAP is catalyzed mainly by CYP3A4, and to a minor extent by CYP3A5 and CYP2C19, as shown in an *in vitro* study [128]. A range of 10 and 38 μg/mL in serum has been reported in patients treated with therapeutic doses [25].

#### 5.7.1. Pharmacokinetics

The influence of *CYP2C19* and *CYP3A5* genotypes on the population clearance estimates of zonisamide were assessed in 99 Japanese patients with epilepsy [12,38]. The zonisamide clearance in *CYP2C19* hetero EMs and PMs was 16% and 30% lower than in homo EMs, respectively. A gene-dose effect was observed for the number of defective *CYP2C19* allele(s), which lends further credence to the involvement of CYP2C19 in the metabolism of zonisamide in patients [12,129]. Conversely, the CYP3A5*3 genotype did not affect the clearance of zonisamide, indicating that the CYP3A5*3 genotypes do not contribute significantly to the zonisamide pharmacokinetics in Japanese patients with epilepsy [12,129].

#### 5.7.2. Pharmacodynamics

We verified the clinical impact of the *CYP2C19* genotype on the effects of zonisamide therapy in 99 Japanese patients as cited above [12]. The median concentration to dose ratios of zonisamide in PMs tended to be higher than in homo EMs [12]. Two out of 27 PMs (7.4%) and 5 out of 79 hetero EMs (6.3%), but none of the 64 homo EMs (0%), developed zonisamide-specific adverse reactions, fever and/or hypohidrosis [12].

## 6. Population Pharmacokinetics of AEDs

The mixed-effect population pharmacokinetic approach permits study of the sources and correlates of variability in blood concentrations between individuals [130]. Because the population pharmacokinetic approach is the optimal methodology for indentifying the magnitude of the effect of the putative factors affecting the variation in pharmacokinetics in patients, a population pharmacokinetic approach makes it possible to determine the dosing regimen to achieve plasma concentrations within a given target range. Moreover, compared with the traditional pharmacokinetic methods, population pharmacokinetics is better suited for analyzing large-scale clinical studies, where only a few samples are available per subject or patient. Table 2 summarizes the evidence for genetic polymorphisms of drug-metabolizing enzymes associated with the population pharmacokinetic model of AEDs. 

As described, we evaluated the associations between the genotypes of *CYP2C9*, *CYP2C19* and/or *CYP3A5* and population clearance estimates of AEDs such as carbamazepine, phenobarbital and zonisamide (Table 2) [12,36,37,38,43]. For example, the phenobarbital clearance in *CYP2C9* hetero EMs was 48% lower than in homo EMs (Table 2) [12,36]. Valproic acid, a known inhibitor of phenobarbital metabolism, and phenytoin, a substrate of CYP2C9 and CYP2C19, were also incorporated into the PPK models (Table 2). Although the *CYP2C19* genotypes were shown to affect the population clearance estimates of phenobarbital in Japanese patients in a previous study [37,114] in which carriers of the defective *CYP2C9* allele(s) were excluded, we observed no effect of the *CYP2C19* genotype on phenobarbital clearance (*P* > 0.05) (Table 2). It should be noted that the number of *CYP2C9* hetero EMs was small (n = 10 in total), and the effects of *CYP2C9* genotypes on the pharmacokinetics of phenobarbital were model-based, and hence the proof of their involvement was indirect [12,36]. However, these results suggest that the *CYP2C9* genotypes may play an important role in the pharmacokinetics of phenobarbital in Japanese patients with epilepsy, and might affect the ethnic differences in the tolerability of phenobarbital therapy (see section 5.5 ) [12,36]. 

**Table 2:** The population pharmacokinetic model of antiepileptic drugs incorporating the drug-metabolizing enzyme polymorphisms.

AED	Final population pharmacokinetic model	Population	Ref.
Carbamazepine	CL (l/h) = 0.17 × (BW/40)^0.11^ × Dose^0.45^ × 1.40^PHT^ × 1.21^PB^ × 1.08^CYP3A5*3/*3^ × e^ηCL^	Japanese	[12,43]
Phenobarbital	CL (l/h) = 0.23 × (BW/40)^0.21^ × 0.52*^CYP2C9^*^ hetero EM^ × 0.68^VPA^ × 0.85^PHT^ × 0.85^SMID^ × (1 + ηCL)	Japanese	[12,36]
	CL (l/h) = 0.0596 × BW^0.367^ × 0.812*^CYP2C19^*^ PM^	Japanese	[37,114]
Phenytoin	V_max_ (mg/day/kg) = 6.07 × (BW/60)^-0.416^ × 0.582*^CYP2C9^*^ hetero EM^ × (1 + ηV_max_)	Japanese	[33]
	K_m_ (μg/mL) = 4.0 × 1.22*^CYP2C19^*^ hetero EM^ × 1.54*^CYP2C19^*^ PM^ × (1 + ηK_m_)		
Valproic acid	CL (l/h) = 0.0951 × (1 + e^0.0267 × (3-G†)^) × 0.0071 × age × e^ηCL^	Chinese	[120]
Zonisamide	CL (l/h) = 1.22 × (BW/44)^0.77^ × Dose^-0.17^ × 0.84*^CYP2C19^*^ hetero EM^ × 0.70*^CYP2C19^*^ PM^ × 1.24^CBZ^ × 1.28^PHT^ × 1.29^PB^ × e^ηCL^	Japanese	[12,38]

AED, antiepileptic drug; V_max_, maximal elimination rate; K_m_, Michaelis-Menten constants; CL, apparent clearance; BW, body weight; Dose, daily dose of each antiepileptic drug; Homo EM, homozygous extensive metabolizers; Hetero EM, heterozygous extensive metabolizers; PM, poor metabolizers; *CYP2C9* hetero EM = 1, *CYP2C9* homo EM = 0; *CYP2C19* hetero EM or *CYP2C19* PM = 1 if one or two *CYP2C19*-defective alleles are carried, respectively, otherwise 0; CYP3A5*3/*3 = 1, otherwise 0; VPA = 1 if valproic acid is co-administered, otherwise 0; PHT = 1 if phenytoin is co-administered, otherwise 0; CBZ = 1 if carbamazepine is co-administered, otherwise 0; PB = 1 if phenobarbital is co-administered, otherwise 0; SMID = 1 if complications of severe or profound mental retardation with significant behavior impairment are presented, otherwise 0; and η = the independent random error distributed normally with the mean zero. ^†^G was 1, 2, and 3, where the genotype was wild type (CYP2C19*1/ *1 combined with CYP2C9*1/*1) , heterozygous (CYP2C19*1/*2 or CYP2C19*1/*3) , and homozygous genotypes (CYP2C19*2/*2 or CYP2C19*2/*3 or CYP2C19*3/*3 combined with CYP2C9*1/*3), respectively.

Meanwhile, the population clearance estimate of zonisamide in *CYP2C19* hetero EMs and PMs was 16% and 30%, respectively, lower than in homo EMs (Table 2) [12,38]. A gene-dose effect was observed for the number of defective *CYP2C19* allele(s), which lends further credence to the involvement of CYP2C19 in zonisamide metabolism in patients [12,38]. Enzyme-inducing AEDs such as carbamazepine, phenytoin and phenobarbital were also incorporated into the population pharmacokinetic models (Table 2). In contrast to CYP2C19, the CYP3A5*3 genotype did not affect the clearance of zonisamide, indicating that CYP3A5*3 genotypes do not have a major impact on the zonisamide pharmacokinetics in Japanese patients with epilepsy. Therefore, the *CYP2C19* genotypes may play an important role in the pharmacokinetics of zonisamide and may affect the development of adverse reactions (see section 5.7 ) [12,38].

These findings suggest that the population pharmacokinetic approach enables us to define relevant genetic factors, together with other non-genetic factors, and to estimate the magnitude of their effects on the variation in pharmacokinetics in patients. Additionally, although the co-expression of drug-metabolizing enzymes and transporters should also be considered [130], population pharmacokinetic modeling in patients can be used to assess the contribution of drug-metabolizing enzymes on the metabolisms of AEDs, and to predict the AED-induced adverse reactions.

**Table 3:** The clinical impact of genetic polymorphisms of drug-metabolizing enzymes on AED therapy.

AED	Genetic polymorphisms	Associated pharmacokinetic or pharmacodynamic parameters	References
Carbamazepine	CYP3A5*3/*3 genotype	Oral clearance	[12,43,44]
	*EPHX1* Try113His and His139Arg	Maintenance dose	[84]
	*GSTM1* null genotype	Mild hepatotoxicity	[88]
Clobazam	*CYP2C19* hetero EMs and PMs	Serum *N*-desmethylclobazam concentration, responder rate	[35]
Lamotrigine	*UGT2B7* -161C>T	Concentration to daily dose ratio	[97]
Phenobarbital	*CYP2C19* PMs	Oral clearance	[37]
	*CYP2C9* hetero EMs	Oral clearance, ethnic differences in tolerability (?)	[12,36]
Phenytoin	*CYP2C9* hetero EMs/PMs and/or *CYP2C19* PMs	Plasma concentration, maintenance dose, CNS toxicity	[12]
	CYP2C9*1B haplotype	Maintenance dose	[104]
	*EPHX1* 113Try/139His haplotype	Risk of craniofacial abnormalities	[58]
Valproic acid	*CYP2C9* hetero EMs and *CYP2C19* PMs	Oral clearance	[120]
	*GSTM1* and *GSTT1* null genotypes	Mild elevation of γ-glutamyltransferase	[125]
Zonisamide	*CYP2C19* hetero EMs and PMs	Oral clearance, zonisamide-specific adverse reactions	[12,38]

AED, antiepileptic drug; *CYP*, *cytochrome P450*; *EPHX1*, *microsomal epoxide hydrolase* gene; *UGT*, *UDP-glucuronosyltransferase*; *GST*, *Glutathione S-transferase*; CNS, central nervous system.

## 7. Conclusions and Future Perspectives

In conclusion, the recent findings indicate that genetic polymorphisms of drug-metabolizing enzymes may have a clinical impact on AED therapy (Table 3). However, at present, screening for these polymorphisms requires intensive genotyping to determine a patient’s optimal dose of AED. Such studies to generate personalized AED therapy are currently quite laborious [7,10]. There are several explanation for the slow progress being made in clinical pharmacogenetics; the most important of which is the fact that gene function is not a constant, but varies depending on environmental factors and the respective gene products themselves [131,132,133]. Another essential problem is the lack of prospective clinical studies assessing the merit of genotyping strategies for AED therapy in large numbers of patients. Therefore, it would be helpful to implement more clinical integration of pharmacogenetic information includings (1) the prospective associations between genetic polymorphisms and the clinical endpoints in the large population; (2) replication of observed associations; (3) studies including various ethnic groups; (4) careful analysis of confounding factors, such as drug-drug interactions and population stratification, and (5) analysis of combinations of genetic polymorphisms of transporters affecting the AED exposure (e.g., organic cation transporter 1 polymorphisms [134]), and other genetic factors affecting the enzyme activity (e.g., transcriptional nuclear factors). The use of population pharmacokinetic modeling incorporating the genotypes of drug-metabolizing enzymes can be one of the most useful tools that can be used to facilitate the determination of individualized dosing regimens in AED therapy, and genetic and non-genetic factors affecting enzyme activity can be used reliably under such models by clinicians in selecting the best AED(s) and dose-adjustment for their patients.
